# Effect of ultra‐high pressure homogenization on microorganism and quality of composite pear juice

**DOI:** 10.1002/fsn3.2906

**Published:** 2022-04-27

**Authors:** Yan Liu, Mengyu Liao, Lei Rao, Liang Zhao, Yongtao Wang, Xiaojun Liao

**Affiliations:** ^1^ 34752 College of Food Science and Nutritional Engineering China Agricultural University Beijing China; ^2^ National Engineering Research Centre for Fruit and Vegetable Processing Beijing China; ^3^ Key Lab of Fruit and Vegetable Processing Ministry of Agriculture and Rural Affairs Beijing China; ^4^ Beijing Key Laboratory for Food Nonthermal Processing Beijing China

**Keywords:** composite juice, microorganism, quality, ultra‐high pressure homogenization

## Abstract

In this study, composite pear juice was processed by ultra‐high pressure homogenization (UHPH) at four different pressures (50, 100, 150, and 200 MPa) with six different temperatures (4, 20, 30, 40, 60, and 80°C), then microorganism and physicochemical and nutritional properties of the samples were investigated. The counts of total aerobic bacteria (TAB) and yeasts and molds (Y&M) were reduced by 0.89–4.72 log10 CFU/ml and 0.40–3.03 log10 CFU/ml after processing, respectively. There was no significant change on total soluble solid and color, but significant decreases of pH and particle size value were observed, and the antioxidant activity, total phenolic content, viscosity, and suspension stability significantly increased in treated samples. Compared to the untreated samples, polyphenol oxidase (PPO) and peroxidase (POD) activity of UHPH‐treated samples varied between 97%–126% and 81%–165%, respectively, indicating that the PPO and POD activities could be inactivated or activated by UHPH. This study introduced proper temperature combined with UHPH could improve the microbial inactivation and the quality of the compound juice.

## Graphical abstract

Effects of temperature and pressure of ultra‐high pressure homogenization (UHPH) on microorganisms and quality of composite juice were explored. UHPH showed the ability to ensure microbiological safety and improve quality of composite juice, and UHPH with proper thermal treatment could improve processing efficiency and avoid adverse effects.

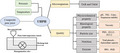

## 1 INTRODUCTION

Fruit juice processed by squeezing the fresh fruit and preserved without being concentrated is labeled as “not from concentrate” (NFC) juice (Grimi et al., 2011). Strong marketing and heightened consumer awareness of the health benefits of consuming phytonutrient‐rich fruits have created the demand for and availability of NFC juices (Beaulieu & Obando‐Ulloa, 2017), because of the fact that it possesses higher nutritional value and more attractive taste compared to its concentrated counterparts.

Pear is one of the most widely used fruits in juice production in China, rich in vitamin, tannic acid, and polyphenol. It has also higher dietary fiber level than most common fruits and vegetables, giving excellent results in the treatment of constipation and intestine inflammation (Brahem et al., 2017). Besides, inverse associations were observed between the intake of pears and cardiovascular disease and all‐cause mortality (Aune et al., 2017), so did breast cancer (Heath et al., 2020). However, with the improvement of consumer level, the taste and nutritional shortcomings of single NFC pear juice are gradually exposed, so it is necessary to develop new composite NFC pear juice.

For many years, heat sterilization has been efficiently applied to process NFC juice. Nevertheless, heat processing has some drawbacks, such as undesirable biochemical and nutritional changes in processed products (color changes, flavor and aroma decreases, and vitamin losses) which may affect the overall quality of the final product (Choi & Nielsen, 2005).

In the last years, efforts have been in the path of studying new food processing technologies that are able to increase the shelf life and ensure food safety without compromising other properties, and ultra‐high pressure homogenization (UHPH, up to 200 MPa) can be placed among these emerging technologies (Saldo et al., 2009).

Ultra‐high pressure homogenization is a novel technology recently studied in the food area to modify the viscous properties of fluids, mainly focusing on the physical changes of the fluid, which has been demonstrated as a valuable tool for microbial inactivation, enzyme inactivation, and improvement of techno‐functional properties of food components (Levy et al., 2021).

During UHPH, the fluid under pressure is forced to pass through a micron‐level hole to produce shear, velocity gradients, turbulence, impingement, cavitation (Campos & Cristianini, 2007), and other effects at the inlet and outlet of the high pressure valve, so as to destroy the cell, achieve microbial inactivation, and change the fluid properties.

Furthermore, some authors also found UHPH‐treated samples get more fruity and with better aroma (Loira et al., 2018). So far, UHPH has been applied to process many continuous fluid foods such as orange juice (Brinez et al., 2006), apple juice (Sauceda‐Galvez et al., 2019), milk (Martinez‐Garcia et al., 2019), cream (Mayta‐Hancco et al., 2019), and mixed fruit beverage (Jayachandran et al., 2016). And the results showed that UHPH could better retain flavor substances and bioactive constituents such as phenols and vitamin.

The heat generated by the dissipation of kinetic during UHPH leads to an increase in temperature, which is made more pronounced by an increase in pressure (Martinez‐Monteagudo et al., 2017), so temperature is an important factor to be concerned about. Most studies of UHPH have focused on the effect of pressure, but few have focused on temperature. Single homogenization treatment cannot achieve good germicidal efficacy; it is necessary to increase the number of homogenization treatment, which reduces the application efficiency of UHPH. Therefore, the microbial inactivation efficiency can be improved by controlling the temperature in the UHPH treatment process. In fact, the passage time through the valve is expected to be extremely short due to the acceleration of the fluid, and the fluid is being subjected to high pressure for usually less than a second (Levy et al., 2021; Martinez‐Monteagudo et al., 2017), the adverse effects of thermal treatment can also be avoided.

The objective of this work was to evaluate the effect of temperature and pressure of UHPH on microorganisms and some qualities of composite NFC pear juice. This study will provide technical support for commercial application of UHHP technique in composite juice processing.

## 2 MATERIALS AND METHODS

### 2.1 Chemicals

2,2‐Diphenyl‐1‐picrylhydrazyl (DPPH), (±)‐6‐hydroxy‐2,5,7,8‐tetramethyl‐ chroman‐2‐carboxylic acid (Trolox), 2,4,6‐tri‐2‐pyridyl‐1,3,5‐triazine (TPTZ), and foline‐phenol were purchased from Shanghai Yuanye Bio‐Technology Co. Anhydrous ferric chloride and anhydrous sodium acetate were purchased from Sinopharma Chemical Reagent Co. Methanol of HPLC grade was purchased from Beijing Chemicals Co. Other chemicals were obtained from Beijing Solarbio Technology Co.

### 2.2 Sample preparation

In this study, the pear variety “Hosui,” harvested at commercial maturity, was obtained from a local store in Hebei (China). The apple variety “Fuji,” harvested at commercial maturity, was obtained from a local store in Shangdong (China). The grapefruit variety “Red Grapefruit,” harvested at commercial maturity, was obtained from a certain import store in Beijing, supplied from South Africa.

Fresh squeezed apple juice and pear juice were obtained from Fuji apple and Hosui pear, respectively, using a juicer (JYZ‐E19 Joyoung Co., Ltd.) and added 0.05% ascorbic acid corresponding to the raw fruit mass to inhibit the enzymatic browning. Fresh squeezed grapefruit were obtained from red grapefruit using a citrus juicer (JE‐601 Yuyao Qidi Electric Appliance Factory). The juices were filtered with eight layers of gauze three times, mixed in proportion, and refrigerated at 4°C until use. According to the previous sensory evaluation, the proportion of composite NFC pear juice was determined as pear juice:apple juice:grapefruit juice = 8:1:1 (w/w/w).

### 2.3 Ultra‐high pressure homogenization treatment

Ultra‐high pressure homogenization treatment was carried out by an ultra‐high pressure homogenizer with APV‐Gaul in type valve (JC‐10NC Guangzhou Juneng biology & technology Co., Ltd.), the flow rate was 10 L/h, and processing time was 1.15 s, the entire homogeneous valve adjusted temperature by water bath, the water bath time from the inlet to the outlet of the sample was about 30 s. Four pressures (50, 100, 150, and 200 MPa) and six temperatures (4, 20, 30, 40, 60, and 80°C) were evaluated. When the water bath temperature reaches the set temperature, the juice is added to the sterilized sample tank, the pressure is controlled by adjusting the knob, then the sample is taken into sterile containers after 30 s of stable pressure, and stored at 4°C until being analyzed. More information about UHPH instrument can be found in the Appendix S1.

### 2.4 Microbiological analysis

The counts of total aerobic bacteria (TAB) and yeasts and molds (Y&M) were estimated using the method described by Wang et al. (2012). An untreated or treated sample was serially diluted with sterile 0.85% NaCl solution, and 1.0 ml of each dilution was plated into duplicate plates of appropriate agar. Nutrient agar (Beijing Solarbio Technology Co., Ltd.) was used for counting the viable TAB cells after incubation at 37°C for 48 ± 2 h. Rose Bengal agar (Beijing Solarbio Technology Co., Ltd.) was used for counting the viable Y&M cells after incubation at 27°C for 72–120 h. After incubation, the colonies were counted. All measurements were made in triplicate.

### 2.5 Measurement of pH and total soluble solids

pH value was measured at 25°C with a Pb‐10 pH meter (Sartorius scientific instruments Co., Ltd).

Total soluble solids (TSS) were measured using an abbe refractometer (DR‐A1, Shanghai precision science instrument co. Ltd) at 25°C, and results were expressed as °Brix. All measurements were made in triplicate.

### 2.6 Measurement of color

Color assessment was conducted at 25°C using a color measurement spectrophotometer (HunterLab Color Quest XE, Hunter Associates Laboratory, Inc.) in the reflectance mode. In this mode, L* (lightness), a* (red to green), and b* (yellow to blue) values were measured. The total color difference (*∆E*) between untreated and treated juices was calculated by applying the formula:

$$\Delta E={\left[{\left(L*‐L{*}_{0}\right)}^{2}+{\left(a*‐a{*}_{0}\right)}^{2}+\;{\left(b*‐\;b{*}_{0}\right)}^{2}\right]}^{1/2}$$

### 2.7 Quantification of total phenols

Ten milliliters of juice was centrifuged at 6155 *g* for 10 min at 4°C (CF16RXⅡ, Hitachi). The supernatant was collected and diluted 8‐fold with distilled water for further analysis.

The total phenols were determined using the Folin–Ciocalteu method described by Cao et al. (2011) with some modifications. An amount of 0.4 ml diluent was mixed with 2 ml Folin–Ciocalteu reagent (previously diluted 10‐fold with distilled water) and 1.8 ml sodium carbonate solution (7.50%), set for 1 h in the dark at room temperature, and then the mixture was immediately measured at 765 nm using a spectrophotometer (UV‐726, Shimadzu). Results were expressed as µg of gallic acid equivalent (GAE) per milliliter juice (µg GAE/ml). The standard curve can be found in Appendix S1.

### 2.8 Antioxidant capacity measurements

#### 2.8.1 Antioxidant capacity determined by stable radical method

Refer to the method of Miller et al. (1995) and modify it slightly. Ten milliliter of juice was centrifuged at 6155 *g* for 10 min at 4°C (CF16RXⅡ, Hitachi) to get the extract. The extract (100 μl) was mixed with 4 ml methanolic determined by stable radical method (DPPH) solution (0.14 mmol/L). The samples were kept in the dark for 45 min at room temperature before measurement of the decrease in the absorption at 517 nm. Determinations were performed using a spectrophotometer (UV‐726, Shimadzu). Trolox solutions within the range of 100–1000 μmol/L were used for calibration. A new Trolox calibration curve was made for each assay. The results were expressed as Trolox equivalents (TE) where one TE equals the net protection produced by one mmol Trolox. The standard curve can be found in Appendix S1.

#### 2.8.2 Antioxidant capacity determined by ferric reducing antioxidant power

Refer to the method of Aljadi and Kamaruddin (2004) and modify it slightly. Freshly prepared ferric reducing antioxidant power (FRAP) solution contained acetate buffer (pH 3.6), 10 mmol/L TPTZ (dissolved in 40 mmol/L HCl), and 20 mmol/L ferric chloride by a ratio of 10:1:1. Four milliliter of FRAP solution was mixed with 100 μl Trolox solution or extract at 37°C. Ten minutes later, the ferric reducing ability of samples was measured by monitoring the increase of absorbance at 593 nm with a spectrophotometer (UV‐726, Shimadzu). The results were expressed as TE. The standard curve can be found in Appendix S1.

### 2.9 Measurement of rheological property

The rheological property of juice was measured by a TA‐1000 rheometer (TA Instruments, Waters Co., Ltd.) using plate (40 mm diameter with a gap of 1000 µm). An amount of 1.5 ml juice was applied at each measurement at 25°C controlled by circulating water in a thermostatic system. In the test, the shear rate was increased from 1 to 100 s^−1^, and three determinations were performed for each treatment.

### 2.10 Measurement of particle size

Particle size was determined using a mastersizer (LS239, Beckman Instruments, Inc). The samples were diluted in distilled water to reach appropriate laser obscuration (9%–13%). The size distribution was characterized by particle size distribution (PSD), the volume‐weighted mean diameter (*D[4,3]*), and the surface‐weighted mean diameter (*D[3,2]*) values. The formulas for *D[4,3]* and *D[3,2]* can be found in Appendix S1.

### 2.11 Measurement of suspension stability

Suspension stability was performed after centrifuging 30 ml juice samples at 1520 *g* for 10 min at 4°C (CF16RXⅡ, Hitachi). The absorbance was measured before and after centrifugation at a wavelength of 660 nm using a spectrophotometer (UV‐726, Shimadzu), set A_0_ and A, and the A/A_0_ ratio was used to characterize suspension stability.

### 2.12 Measurements of polyphenol oxidase and peroxidase activities

The extraction solution consisted of a 0.2 mol/L phosphate buffer (pH = 6.5) containing 4% (w/v) polyvinylpolypyrrolidone (PVPP). The composite juice and extraction mixture (8 g: 4 ml, m/v) were shaken uniformly and centrifuged at 6155 *g* for 15 min at 4°C (CF16RXⅡ, Hitachi). The supernatant was used to determine PPO and POD activity.

For the PPO assay, 1 ml supernatant was added to 2 ml of 0.2 mol/L phosphate buffer (pH = 6.5) containing 0.5 mol/L catechol. The absorbance was measured at 420 nm for 1 min (scanning interval was 0.1 s) using a UV‐visible spectrophotometer (UV‐1800, Uniko instrument co., Ltd). The analysis was carried out in triplicate.

For the POD assay, 1 ml supernatant was added to 0.2 ml of 1.5% hydrogen peroxide (v/v) and 2.2 ml of 0.2 mol/L phosphate buffer (pH = 6.5) containing 1% guaiacol (v/v). The absorbance was measured at 475 nm (scanning interval was 0.1 s) for 1 min using a UV‐visible spectrophotometer (UV‐1800, Uniko instrument co., Ltd). The analysis was carried out in triplicate.

The residual activity for both PPO and POD enzymes was calculated according to:

$$\text{RA}\left(\%\right)\;=\frac{A}{A_{0}}\times 100$$

where *A* is the activity of the UHPH‐treated juice and *A*_0_ is the activity of the untreated juice.

### 2.13 Statistical analysis

Statistical analysis was performed using the SPSS Statistics 22 software program. The correlation coefficient (*R* ^2^) and *p*‐value were used to show correlations and significances. Values of *p* < .05 were considered statistically significant.

## 3 RESULTS AND DISCUSSION

### 3.1 Microbiological analysis

The initial counts of TAB and Y&M in untreated juice were 4.72 and 3.03 log_10 CFU_/ml, respectively. In this study, the factors affecting the germicidal efficacy were pressure and temperature.

**FIGURE 1:**
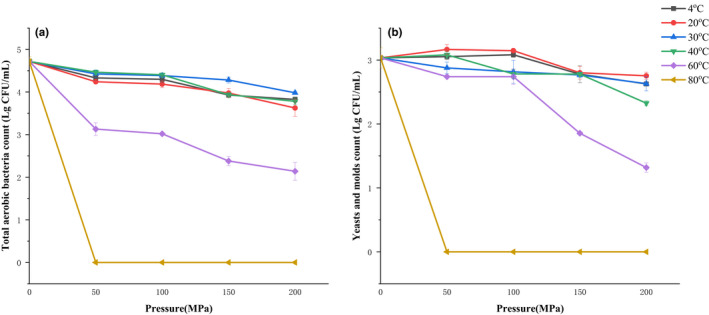
Effect of ultra‐high pressure homogenization (UHPH) treatments on the total aerobic bacteria (a) and yeast and mold (b) of composite pear juice

As shown in Figure 1, when the temperature was the same, the count of TAB decreased significantly with the increase of pressure, that was, increasing the pressure significantly improved the microbial inactivation efficacy. Several studies in UHPH application had been made to date and demonstrate the reduction of microbial loads in different food matrices, e.g., milk and different fruit juices (Dong et al., 2021; Roig‐Sagues et al., 2015).

The mechanisms by which UHPH inactivates microorganisms are shear stress, cavitation, and high‐speed turbulence (Georget et al., 2014). In microorganisms, these physical phenomena could increase the permeability or rupture of the cell membrane causing cell death (Reverter‐Carrion et al., 2018), and the increase of pressure will increase the strength of these physical phenomena. Similarly, when the pressure was constant, increasing the temperature could significantly improve the microbial inactivation efficacy. Moreover, homogeneous treatments resulted in the reduction of TAB to a level below the detection limit in all stated pressure at 80°C.

These results indicated that, compared to increasing pressure, increasing temperature had a more significant effect on bactericidal efficacy. According to Roig‐Sagues et al. (2015), UHPH treatments hardly affected the spore counts at the lowest temperature (20°C), but significant reductions were observed when the temperature raised to 60°C. Amador Espejo et al. (2014) and Benjamin and Gamrasni (2020) also reported that increasing the temperature was more efficient in the research of milk and pomegranate juices. Therefore, the temperature improved the rate of microorganism inactivation in a synergic effect with the pressure applied, enhancing the effectiveness of the treatment (Amador Espejo et al., 2014). Not only can high temperature destroy cell membranes, but also can make the flow pattern of the fluid exhibit more turbulence, resulting in the enhancement of microbial inactivation due to increasing cavitation of fluid according to Diels et al. (2004). Pathanibul et al. (2009) also reported that when UHPH treatments are below 200 MPa, the inactivation obtained is mainly due to mechanical effects. However, when homogenization pressures are greater than 250 MPa, the homogenization‐induced thermal contributions were predominant (Kumar et al., 2009; Taylor et al., 2007).

The result of Y&M was consistent with that of TAB. Welti‐Chanes et al. (2009) demonstrated that a significant difference is observed for mesophiles and yeasts plus molds in juice previously after heating at same pressures. Similarly, significant reductions in yeasts and molds at higher pressure were obtained (Szczepanska et al., 2021).

According to Chinese Food Safety Standards‐Drinks GB 7101–2015, the count of TAB in a beverage is limited to 3.0 log_10_ CFU/ml, and the limits for yeasts and molds are 1.30 log_10_ CFU/ml and 1.69 log_10_ CFU/ml, respectively. When temperature was 80°C, the composite NFC pear juice after UHPH treatments met microbiological safety standards. Therefore, UHPH with proper thermal treatment showed better microbial inactivation ability to ensure the safety of composite NFC pear juice.

### 3.2 pH, TSS, and color analysis

As shown in Table 1, at 60 and 80°C, all treated samples showed significant reductions in pH values, and there were no significant differences in pH values at other temperatures. Meanwhile, the increase of pressure had no significant effect on pH value at the same temperature. The reason may be that the homogenization effect destroys the cells, dissolves organic acids and other substances, that is, the extraction effect in disguised form, and reduces the pH value. Similar results were reported in the researches of apricot juice (Patrignani et al., 2013) and blackcurrant juice (Kruszewski et al., 2021). Moreover, this result indicated that increasing the temperature could accelerate this extraction effect. Other researches also reported increases in pH of mango juice and mixed juiced because of releasing cell components (Wellala et al., 2020; Zhou et al., 2017). Therefore, effects of UHPH on pH of juice could depend on food matrices and treatments.

**TABLE 1:** Effect of ultra‐high pressure homogenization (UHPH) treatments on pH, TSS, and color of composite pear juice

		pH	TSS (°Brix)	Color			
				L*	a*	b*	*ΔE*
Untreated		3.86 ± 0.04^b^	11.27 ± 0.38^de^	28.71 ± 0.63^d^	0.14 ± 0.62^c^	0.72 ± 0.66^c^	0
4°C	50 MPa	3.81 ± 0.00 cd	11.87 ± 0.06^a^	28.84 ± 0.07^d^	0.01 ± 0.20^c^	0.75 ± 0.38^c^	0.73
	100 MPa	3.82 ± 0.01^c^	11.70 ± 0.00^b^	28.51 ± 0.47^d^	0.13 ± 0.15 cd	1.06 ± 0.16^b^	0.50
	150 MPa	3.81 ± 0.01 cd	11.60 ± 0.00^c^	29.13±0.09^c^	0.05 ± 0.20 cd	1.09 ± 0.20^b^	0.93
	200 MPa	3.82 ± 0.00^c^	11.63 ± 0.06^c^	28.71 ± 0.10^d^	0.09 ± 0.14^c^	1.06 ± 0.10^b^	0.53
20°C	50 MPa	3.81 ± 0.00 cd	11.00 ± 0.00^f^	29.86 ± 0.22^ab^	0.13 ± 0.08^c^	0.54 ± 0.26 cd	0.40
	100 MPa	3.80 ± 0.01^def^	11.00 ± 0.06^f^	30.51 ± 0.52^a^	0.10 ± 0.17^c^	0.88 ± 0.19^bc^	0.94
	150 MPa	3.81 ± 0.01 cd	11.00 ± 0.06^ef^	29.77 ± 0.23^b^	0.40 ± 0.15^bc^	0.77 ± 0.18^c^	0.41
	200 MPa	3.81 ± 0.01^cde^	11.03 ± 0.06^ef^	29.72 ± 0.53^b^	0.49 ± 0.13^bc^	0.83 ± 0.17^c^	0.62
30°C	50 MPa	3.89 ± 0.01^a^	11.87 ± 0.06^a^	29.40 ± 0.58^bc^	−0.24 ± 0.19^d^	1.60 ± 0.57^ab^	0.81
	100 MPa	3.89 ± 0.01^a^	11.30 ± 0.00^d^	29.70 ± 0.36^b^	−0.44 ± 0.13^d^	1.73 ± 0.58^ab^	0.93
	150 MPa	3.89 ± 0.00^a^	11.40 ± 0.10^d^	29.41 ± 0.33^bc^	0.20 ± 0.35 cd	1.80 ± 0.32^a^	0.74
	200 MPa	3.89 ± 0.01^a^	11.43 ± 0.06^d^	30.24 ± 0.34^a^	0.00 ± 0.20 cd	1.89 ± 0.32^a^	1.36
40°C	50 MPa	3.89 ± 0.01^a^	11.37 ± 0.10^d^	28.33 ± 0.48^d^	0.17 ± 0.21^c^	0.65 ± 0.10^c^	0.63
	100 MPa	3.88 ± 0.01^a^	11.40 ± 0.00^d^	28.59 ± 0.51^d^	0.12 ± 0.06^c^	0.76 ± 0.06^c^	0.62
	150 MPa	3.88 ± 0.00^a^	11.40 ± 0.06^d^	28.51 ± 0.51^d^	0.13 ± 0.04^c^	0.02 ± 0.12^d^	0.64
	200 MPa	3.88 ± 0.01^a^	11.67 ± 0.12^bc^	28.77 ± 0.24^d^	0.15 ± 0.13^c^	0.89 ± 0.34^bc^	0.83
60°C	50 MPa	3.80 ± 0.00^def^	11.17 ± 0.06^e^	29.07 ± 0.25 cd	1.20 ± 0.25^a^	0.01 ± 0.37^d^	0.82
	100 MPa	3.79 ± 0.00^efg^	11.17 ± 0.06^e^	28.73 ± 0.06^d^	1.26 ± 0.27^a^	−0.14 ± 0.57^d^	0.66
	150 MPa	3.78 ± 0.00^gh^	11.10 ± 0.00^ef^	28.73 ± 0.06^d^	1.26 ± 0.27^a^	−0.14 ± 0.57^d^	0.66
	200 MPa	3.78 ± 0.01^fg^	11.23 ± 0.06^de^	29.03 ± 0.25^d^	1.38 ± 0.28^a^	0.06 ± 0.23^d^	0.81
80°C	50 MPa	3.77 ± 0.00^hi^	11.17 ± 0.06^e^	29.76 ± 0.25^b^	1.38 ± 0.28^a^	0.06 ± 0.23^d^	0.81
	100 MPa	3.76 ± 0.01^i^	11.13 ± 0.06^e^	29.50 ± 0.36^bc^	1.12 ± 0.18^a^	−0.09 ± 0.29^d^	0.52
	150 MPa	3.76 ± 0.00^i^	11.23 ± 0.06^de^	29.65 ± 0.20^b^	1.19 ± 0.13^a^	0.20 ± 0.13^d^	0.31
	200 MPa	3.76 ± 0.00^i^	11.27 ± 0.06^de^	29.71 ± 0.05^b^	1.14 ± 0.11^a^	0.01 ± 0.16^d^	0.21

Note

All data were the means ± SD, *n* = 3.

Values with different letters within one column are significantly different (*p* ˂ .05).

There was no significant difference in TSS after UHPH treatment. This result agreed with that obtained by Velázquez‐Estrada et al. (2019), Benjamin and Gamrasni (2020), and Wellala et al. (2020), in which the TSS of orange juice, pomegranate juice, and mixed juices were not changed after UHPH processing.

Color differences were characterized by L*, a*, and b* values, and results were also shown in Table 1.

Ultra‐high pressure homogenization treatment significantly increased L* values at 20, 30, and 80°C, causing brighter color. In other temperatures, there were no significant differences. There was no significant increase but a trend to increase as pressure increased. Juice lightness increased probably due to the fragmentation of suspended particles which manifested through the size reduction and shape change (Kruszewski et al., 2021). UHPH reduced the particle size, and the small size particles had higher abilities to scatter light (Calligaris et al., 2012),resulting in increased L* values. Saldo et al. (2009) reported increases in L* value of apple juice at 200/300 MPa and attributed it to the presence of smaller size particles.

For a*, UHPH treatment significantly increased a* values at 60 and 80°C. For b*, UHPH treatment significantly increased b* values at 4 and 30°C; however, b* values decreased significantly at 60 and 80°C. Increasing pressure had no significant effect on a* and b* values. The breakdown of the cells caused the dissolution of pigments, which could be responsible for the improvements of a* and b* values. Karacam et al. (2015) reported that the effect of UHPH on the juice's red color can be attributed to the higher level of anthocyanins release under UHPH shearing forces. Liu et al. (2019) and Kaneiwa Kubo et al. (2013) also reported similar increase of a* and b* values. In addition, the increases of b* values may be related to the increase in enzyme activity associated with browning, such as polyphenol oxidase (PPO) and peroxidase (POD). Therefore, the decrease of activities of browning‐related enzymes (Figure 4) in high temperature could result in the reduction of b* values.

The *∆E*, as an indicator of total color difference, reflects a noticeable visual difference when the value is not less than 2. In all samples, *ΔE* values increased but also were lower than 2, which indicated that there were no noticeable changes observed in the color of treated samples in comparison to the untreated ones. Therefore, UHPH treatment had a good ability to maintain the color of the juice. This phenomenon has also been proposed in researches of mango juice by Guan et al. (2016) and pomegranate juice by Benjamin and Gamrasni (2020).

### 3.3 Total phenols and antioxidant capacity analysis

As shown in Table 2, the total phenolics content of composite pear juice was significantly decreased at 4°C while increased at other temperatures after UHPH. At the same temperature, with the increase of pressure, the total phenol contents of UHPH‐treated samples increased significantly.

**TABLE 2:** Effect of ultra‐high pressure homogenization (UHPH) on total phenols, antioxidant capacity, *D[4,3]*, *D[3,2]*, and suspension stability of composite pear juice

		Total phenols (µg GAE/ml)	DPPH (mmol/L Trolox)	FRAP (mmol/L Trolox)	*D[4,3]* /µm	*D[3,2]* /µm	Suspension stability
Untreated		25.86 ± 0.82^g^	0.139 ± 0.016^cde^	0.237 ± 0.009^d^	12.46 ± 0.26^a^	8.07 ± 0.13^a^	0.19 ± 0.06^m^
4°C	50 MPa	17.63 ± 0.52^k^	0.107 ± 0.003^lm^	0.173 ± 0.003^h^	1.43 ± 0.00^b^	1.02 ± 0.00^fg^	0.37 ± 0.02^kl^
	100 MPa	19.92 ± 0.25^j^	0.114 ± 0.003^ijkl^	0.199 ± 0.007^fg^	1.23 ± 0.00^c^	0.94 ± 0.00^ghi^	0.60 ± 0.27^cdefg^
	150 MPa	20.60 ± 0.65^j^	0.120 ± 0.003^ghijkl^	0.199 ± 0.003^fg^	1.14 ± 0.00^cde^	0.89 ± 0.00^ij^	0.65 ± 0.01^bcdef^
	200 MPa	22.74 ± 1.04^i^	0.129 ± 0.003^defghi^	0.208 ± 0.003^ef^	1.09 ± 0.00^de^	0.87 ± 0.00^ij^	0.70 ± 0.02^bcd^
20°C	50 MPa	29.04 ± 0.45^ab^	0.139 ± 0.005^cde^	0.163 ± 0.006^h^	1.42 ± 0.00^b^	1.00 ± 0.00^fgh^	0.61 ± 0.04^cdefg^
	100 MPa	28.25 ± 0.75^bc^	0.141 ± 0.004 cd	0.195 ± 0.007^fg^	1.24 ± 0.00^c^	0.93 ± 0.00^ghi^	0.66 ± 0.02^bcde^
	150 MPa	29.04 ± 0.45^ab^	0.156 ± 0.004^ab^	0.193 ± 0.003^g^	1.24 ± 0.01^c^	0.92 ± 0.01^hi^	0.71 ± 0.02^bcd^
	200 MPa	29.84 ± 0.65^a^	0.161 ± 0.002^a^	0.202 ± 0.003^fg^	1.10 ± 0.00^de^	0.86 ± 0.00^ij^	0.85 ± 0.08^a^
30°C	50 MPa	24.81 ± 0.49^h^	0.132 ± 0.005^cdefgh^	0.206 ± 0.006^efg^	1.10 ± 0.06^de^	1.67 ± 0.17^c^	0.29 ± 0.02^lm^
	100 MPa	24.70 ± 0.35^h^	0.134 ± 0.004^cdef^	0.232 ± 0.006^d^	1.19 ± 0.02 cd	1.82 ± 0.02^b^	0.45 ± 0.03^ijk^
	150 MPa	26.66 ± 0.28^fg^	0.144 ± 0.001^bc^	0.236 ± 0.005^d^	1.11 ± 0.01^de^	1.62 ± 0.01^c^	0.66 ± 0.01^bcde^
	200 MPa	27.24 ± 0.27^def^	0.146 ± 0.004^bc^	0.236 ± 0.004^d^	0.96 ± 0.00^f^	1.30 ± 0.01^d^	0.69 ± 0.01^bcde^
40°C	50 MPa	23.50 ± 0.92^i^	0.095 ± 0.009^m^	0.168 ± 0.006^h^	1.09 ± 0.00^e^	1.62 ± 0.00^c^	0.47 ± 0.01^hijk^
	100 MPa	26.00 ± 0.51^g^	0.110 ± 0.000^kl^	0.237 ± 0.008^d^	0.96 ± 0.01^f^	1.31 ± 0.01^d^	0.58 ± 0.02^defgh^
	150 MPa	26.76 ± 0.44^fg^	0.139 ± 0.027^cde^	0.231 ± 0.005^de^	0.85 ± 0.01^g^	1.10 ± 0.02^e^	0.56 ± 0.03^efghi^
	200 MPa	26.73 ± 0.66^fg^	0.128 ± 0.006^defghij^	0.233 ± 0.003^d^	0.81 ± 0.03^gh^	1.04 ± 0.04^ef^	0.58 ± 0.05^cdefgh^
60°C	50 MPa	26.55 ± 0.17^fg^	0.121 ± 0.003^fghijkl^	0.217 ± 0.002^e^	0.77 ± 0.00^ghi^	0.94 ± 0.01^ghi^	0.42 ± 0.02^jk^
	100 MPa	28.18 ± 0.33^bcd^	0.133 ± 0.003^cdefg^	0.235 ± 0.001^d^	0.75 ± 0.00^ghi^	0.93 ± 0.00^ghi^	0.50 ± 0.02^ghij^
	150 MPa	28.14 ± 0.38^bcd^	0.122 ± 0.004^fghijk^	0.237 ± 0.001^d^	0.74 ± 0.00^hi^	0.90 ± 0.00^ij^	0.51 ± 0.04^fghij^
	200 MPa	29.59 ± 0.49^a^	0.137 ± 0.006^cde^	0.246 ± 0.004^d^	0.70 ± 0.00^i^	0.82 ± 0.00^j^	0.62 ± 0.07^cdefg^
80°C	50 MPa	26.51 ± 0.29^fg^	0.114 ± 0.005^jkl^	0.311 ± 0.013^c^	0.83 ± 0.00^gh^	1.07 ± 0.00^ef^	0.62 ± 0.01^cdefg^
	100 MPa	27.05 ± 0.10^ef^	0.118 ± 0.008^hijkl^	0.314 ± 0.012^c^	0.73 ± 0.00^hi^	0.90 ± 0.01^ij^	0.69 ± 0.09^bcde^
	150 MPa	27.82 ± 0.29^cde^	0.121 ± 0.004^fghijkl^	0.344 ± 0.017^b^	0.69 ± 0.00^i^	0.81 ± 0.00^j^	0.77 ± 0.11^ab^
	200 MPa	27.78 ± 0.22^cde^	0.126 ± 0.003^efghij^	0.366 ± 0.020^a^	0.78 ± 0.04^ghi^	0.99 ± 0.07^fgh^	0.71 ± 0.03^bc^

Note

All data were the means ± SD, *n* = 3.

Values with different letters within one column are significantly different (*p* ˂ .05).

The change of total phenol content may be related to the homogeneous effect and enzymatic reaction. With the increase of pressure and temperature, the fragmentation effect of UHPH on cells and the dissolution of polyphenols increased. This phenomenon can be explained by the formation of derivatives of already present phenolic compounds and/or new ones through the hydrolysis and depolymerization of complexes induced directly by high mechanical forces during the UHPH process, as well as the enhanced extraction of these compounds from the mechanically destroyed cells (Kruszewski et al., 2021). Similarly, the increase of total phenols was obtained in apple juice (Suarez‐Jacobo et al., 2011) and mango juice (Guan et al., 2016) during UHPH treatment after increasing pressure and temperature.

The change of enzyme activity related to enzymatic reaction such as PPO and POD also had a significant effect on the content of total phenol. At 4°C, the increasing of PPO activity (Figure 4) which promoted the decomposition of phenolic compounds (Gahler et al., 2003) was the main reason why total phenols content was lower than untreated group significantly. Similarly, it was also observed in apple juice (Suarez‐Jacobo et al., 2011). With the increase of temperature, the decrease of total phenol content at 30°C may be related to the activation of POD (Figure 4). Moreover, heat also can speed the decomposition process of phenolic compounds (Karacam et al., 2015); that is why there was a significant decline in treated samples at 80°C compared with samples at 60°C.

Generally, there is a dynamic equilibrium between homogenization and enzymatic reaction. At low temperature and low pressure, the total phenol content was decreased due to the dominance of enzymatic reaction, while with the increase of pressure or temperature, the homogenization effect was strengthened, which increased the total phenol content.

The antioxidant capacity of composite juice was evaluated using DPPH and FRAP assays (Table 2). For FRAP antioxidant capacity, the values of samples treated at 4 and 20°C significantly decreased while the values of samples treated at 80°C significantly increased in contrast with that of untreated samples. This result indicated that increasing temperature could promote the improvements of the FRAP antioxidant capacity at the same pressure. Likewise, increasing pressure could lead to similar results.

The result of DPPH antioxidant capacity was similar with that of FRAP antioxidant capacity; however, the DPPH antioxidant capacity showed a significant decrease at 40 and 80°C.

The results via two assays were different. This might be related to the different principles which give different results (Ryan & Prescott, 2010; Thaipong et al., 2006). DPPH method focuses on the scavenging ability of one free radical, while FRAP method focuses on the total reducing ability of the sample. Therefore, it is necessary to use different methods to elucidate the total antioxidant capacity of a sample.

The change of antioxidant capacity was similar to the change of total phenols content. It has been reported that the antioxidant capacity of fruit juice is closely related to the bioactive components (total phenols, etc.) in fruit juice (Gardner et al., 2000; Gunduz & Ozdemir, 2014). Studies had shown that phenolic compounds exhibit a strong antioxidant capacity that contributes to the total antioxidant capacity, which was observed in mulberry juice (Yu et al., 2014) and apple juice (Suarez‐Jacobo et al., 2011), and the release of more content of polyphenols during intensive cell disruption caused by homogenization enhanced the antioxidant capacity. Therefore, the change of antioxidant capacity is related to the change of polyphenols caused by homogenization effect and enzymatic reaction.

### 3.4 Rheological property analysis

**FIGURE 2:**
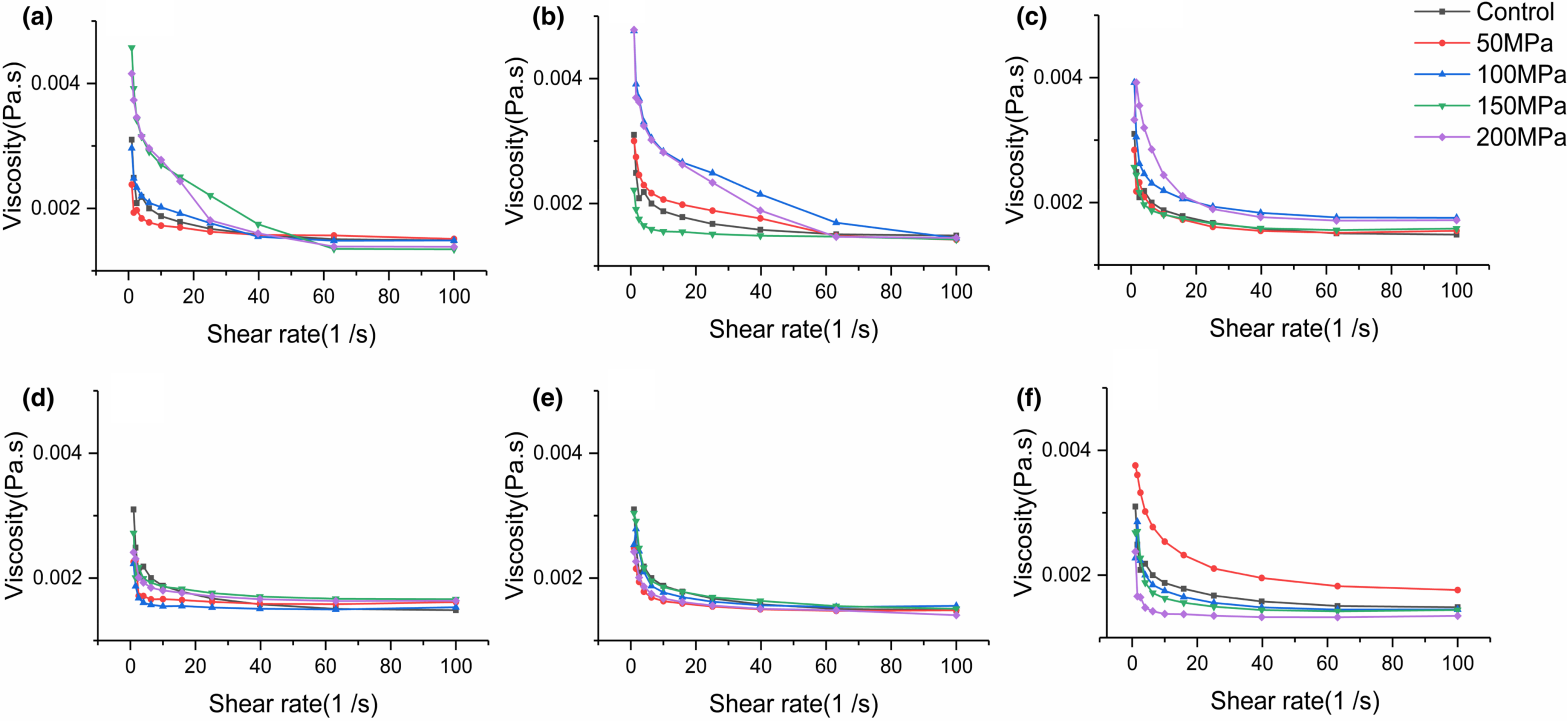
Effect of ultra‐high pressure homogenization (UHPH) treatments on rheological properties of composite pear juice. The treatment temperature is 4°C (a), 20°C (b), 30°C (c), 40°C (d), 60°C (e), and 80°C (f)

As shown in Figure 2, with the increase of shear rate, the viscosity of composite juice gradually decreased and presented the characteristics of shear thinning, which was a pseudo‐plastic fluid. Moreover, the fluid type of the sample was not changed by UHPH treatment.

Compared to untreated sample, fluid viscosity of the sample significantly increased after UHPH treatments at 4, 20, and 30°C with the increase of pressure. When temperature reached 40 and 60°C, fluid viscosity had no significant difference with untreated sample. However, fluid viscosity of the sample processed at 80°C decreased with the increase of pressure.

Augusto et al. (2012) reported a similar result in the study on tomato juice treated at 150 MPa, and temperature was lower than 40°C. It was explained that the homogenization treatment led to increased broken suspended particles, and the interaction between particles enhanced, thus the observed behavior showed an increase in apparent viscosity. Similar result was also observed by Bayod et al. (2008). Furthermore, UHPH could lead to pectin chain fracture degradation (Bengtsson & Tornberg, 2011) and increase interactions of pectin polymers (Wellala et al., 2020), resulting in increased viscosity. Huang et al. (2020) reported the increased viscosity of sugar beet pulp suspension could be attributed to the internal pectin exposure caused by homogenization treatment. Karacam et al. (2015) suggested a higher viscosity in strawberry juice may be due to activation of the pectin methylesterase. Zhou et al. (2017) suggested that the increase in apparent viscosity could be caused by the increase in solubility of high‐molecular‐weight carbohydrates such as starch and pectin.

High temperature resulted in a decrease in the viscosity of juice. Nindo et al. (2005) reported that as the temperature increases, the movement of molecules is promoted, and the average volume occupied by each molecule is increased, so that the viscosity of the liquid is reduced. In addition, the homogenization effect increased further with the increase of temperature, and the breakdown of intermolecular interactions resulted in a decrease in viscosity. When the effect of temperature exceeded that of pressure, the viscosity of the fluid was expressed as decreasing or no significant difference with untreated group.

In summary, similar to the change of total phenol content, the effect of pressure and temperature on viscosity is a dynamic equilibrium process. The influence of pressure on viscosity is dominant at low temperature (4–30°C), resulting in an increase in viscosity, while the influence of temperature on viscosity is dominant at high temperature (40–80°C), resulting in a decrease in viscosity.

### 3.5 Particle size analysis

**FIGURE 3:**
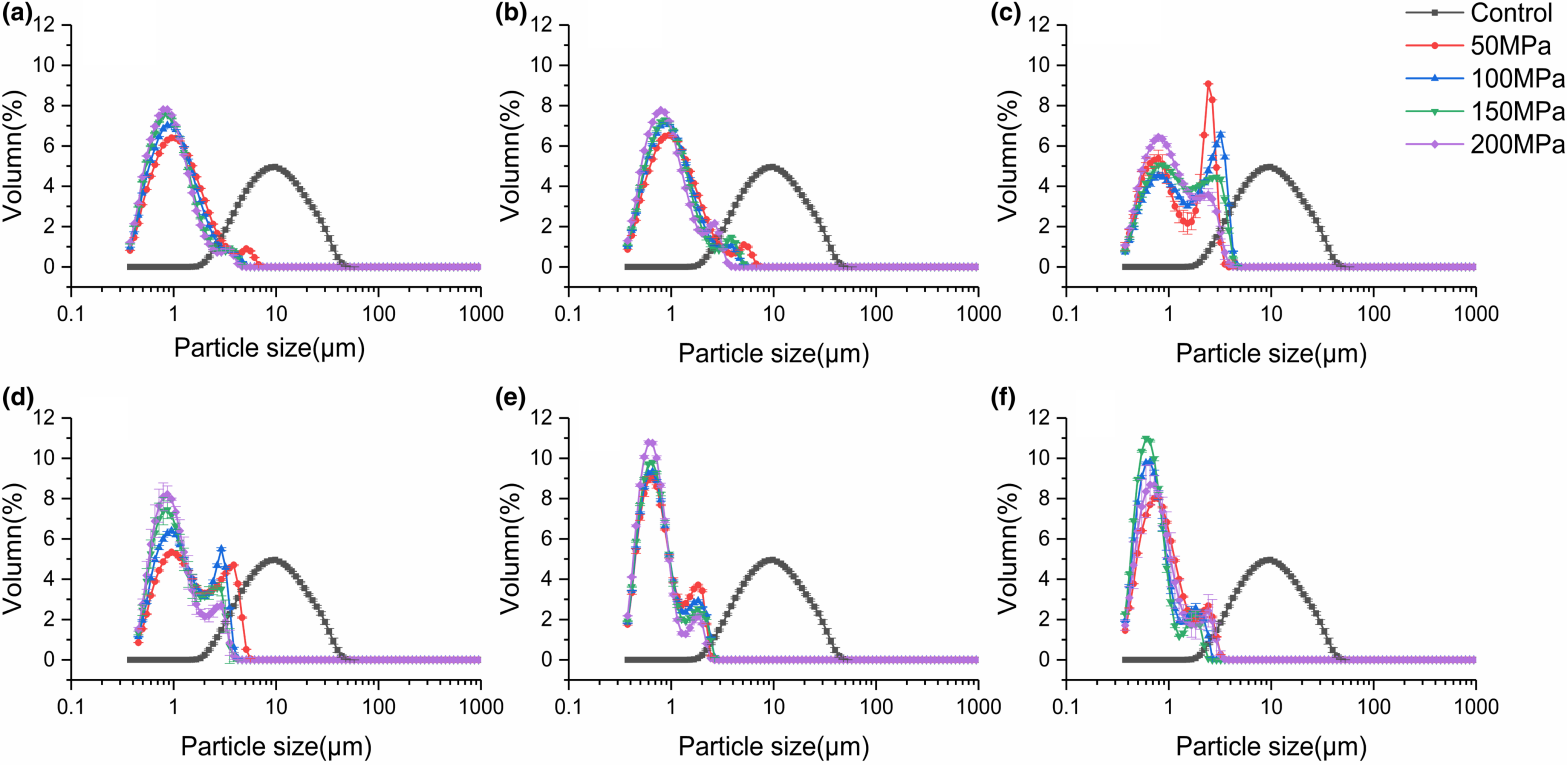
Effect of ultra‐high pressure homogenization (UHPH) treatments on particle size of composite pear juice. The treatment temperature is 4°C (a), 20°C (b), 30°C (c), 40°C (d), 60°C (e), and 80°C (f)

As shown in Figure 3, the particle size distribution (PSD) of untreated NFC pear juice was 1–100 μm, while that of UHPH treatment was 0.1–10 μm. With the increase of pressure, the PSD peak shifted to the left, and the particle size of juice decreased significantly, and the small particle size increased gradually. Moreover, the peak of PSD became narrower and higher, which meant that the particles are distributed more evenly. Similar results were observed by previous researchers for mixed juice (Wellala et al., 2020) and tomato pulp (Panozzo et al., 2013).

However, as the pressure increased, the changes in particle size were less pronounced. The change in particle diameter between 50 and 200 MPa was not distinct from that between 0 and 50 MPa. Augusto et al. (2012) reported it and described that the effect of homogenization pressure on the disruption of suspended particles seems to follow an asymptotic behavior. The same conclusion was drawn, and it was pointed out that the changes in particle size caused by increased pressure at high pressure were smaller than that caused by increased pressure at low pressure(Yu et al., 2021). In fact, this can even be observed in the *D[3,2]* and *D[4,3]* values in Table 2. The reason may be that smaller fragments are less likely to break up during processing than larger fragments or even whole cells.

Temperature increase had no significant effect on distribution range, but had effect on peak shape. The PSD changed from single peak to double peak, the peak value representing larger particles (particle size greater than 1 μm) was lower and the particle size ranges of the two peaks got closer. Leite et al. (2017) also reported it in the orange juice study.

When temperature was low (4–20°C), the increase of temperature had no significant effect on PSD, but when the temperature reached 30 and 40°C, the number of particles in the range of 0.1–1 μm decreased in the range of 1–10 μm increased significantly. However, when the temperature reached 60°C and 80°C, the changes in these two particle size ranges were opposite. The temperature increase promoted the protein denaturation and aggregation, thus increasing the particle size (Augusto et al., 2012). Therefore, at moderate temperature, the effect of temperature on the change of PSD was more significant. At higher temperature, this effect is not obvious compared with the enhanced homogenization effect.

It was reported that *D[3,2]* is more influenced by the smaller particles, while the *D[4,3]* is more influenced by the larger ones (Yu et al., 2016). As shown in Table 2, *D[3,2]* and *D[4,3]* decreased significantly after UHPH treatment. This indicated that after UHPH treatment, the large particles significantly reduced and the small particles were significantly increased, which was consistent with the results of PSD.

For the untreated sample, the *D[4,3]* value was almost 1.5 times higher than the *D[3,2]* value, confirming the contents of larger particles were a little higher than that of smaller ones. After UHPH treatment, the value of *D[4,3]* was close to the value of *D[3,2]*; the peak distributed in larger size could be residual particles of large particles after crushing, which indicated that the subsequent disruptions were preferentially of the larger particles.

*D[4,3]* and *D[3,2]* decreased significantly as the pressure increased, as did *D[4,3]* with temperature. However, with the increase of temperature, *D[3,2]* decreased significantly at 4–20°C and 40–80°C and increased significantly at 30–40°C.

This indicated that with the increase of temperature and pressure, the degree of particles breakage increased. However, with the increase of temperature, the aggregation of small particles existed at 30–40°C, while this effect diminished with the further increase of temperature, which was consistent with the results of PSD.

### 3.6 Suspension stability analysis

As shown in Table 2, the suspension stability of the homogenized samples (0.29–0.85) was significantly higher than that of untreated sample (0.19), which indicated that UHPH treatment significantly improved the suspension stability of juice. Velázquez‐Estrada et al. (2019) also reported that orange juice treated by UHPH presented a good cloud stability.

The suspension stability increased significantly with the increased pressure. The increase of pressure made the juice more and more strongly shear and impingement, and the particles became smaller and smaller, thus improving the suspension stability of the juice. Similar results were reported in the research of carrot beverage by Liu et al. (2019) and carrot beverage by Yu et al. (2021). However, with the increase of temperature, the suspension stability showed a trend of first increase, then decrease and then increase, which was related to the change of particle aggregation and fragmentation, and corresponded to the results of particle size.

### 3.7 Polyphenol oxidase and peroxidase activity analysis

**FIGURE 4:**
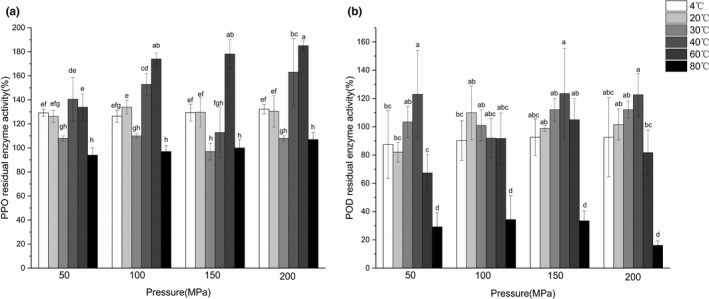
Effect of ultra‐high pressure homogenization (UHPH) on polyphenol oxidase (PPO) (a) and peroxidase (POD) (b) activity of composite pear juice

As shown in Figure 4a, most of UHPH treatments showed activation effect on PPO activity. At the same temperature, PPO activity was significantly increased with the increase of pressure. Similar results were observed in the researches of apple juice by Bot et al. (2018) and pear juice by Liu et al. (2009). This observation might relate to the increase of phenolic compounds and conformational changes of PPO. The change of substrate concentration (phenolic compounds) led to the change of enzyme activity (Balasundram et al., 2006; Nokthai et al., 2010). UHPH induced conformational changes of enzymes that may expose active sites, increasing its activity (Martinez‐Monteagudo et al., 2017). Secondary structures such as *α*‐helix, *β*‐sheet, *β*‐turn, and random roil changed to a certain extent, which resulted in activation of PPO (W. Liu et al., 2009). Another possible reason may be the release of enzymes enclosed in plant vacuoles caused by UHPH and as a result of this phenomenon an increase in the measured activity (Szczepanska et al., 2022). Sauceda‐Galvez et al. (2021) found that PPO in cloudy apple juice increased its activity by 87% after 200 MPa UHPH, while there was no PPO activity detected at 300 MPa UHPH. If the pressure increased further, the enzyme could be inactivated.

Under the same pressure treatment, PPO activity of samples fluctuated with the increase of temperature. PPO activity treated at 4, 20, 40, and 60°C, except 30 and 80°C, was significantly higher than untreated samples. In addition, PPO activity increased significantly with increasing temperature in the range of 40–60°C. However, high temperature (80°C) induced inactivation of PPO, which might be due to the folding or unfolding of PPO molecules induced by high temperature (Castro et al., 2008; Iqbal et al., 2019). Leite Júnior et al. (2021) also reported that there were conformational changes of PPO induced by temperature, which resulted in activation of PPO.

Above all, the observation can be mainly related to the substrate concentration and conformational changes of PPO which are induced by pressure and temperature. With the increase of temperature, dominant factor changed. The increased substrate concentration led to the increase of PPO activity at low temperature, while the conformational changes of PPO led to inactivation at high temperature. Meanwhile, high pressure also could cause conformational changes of enzyme, and conformational changes induced by temperature was another dominant factor.

As shown in Figure 4b, most of the UHPH treatments exhibited inactivation effect on POD activity. There was no significant difference between treated samples at same temperature with the increase of pressure. Yi et al. (2018) also found similar result in cloudy apple juice added kiwifruit puree under HPH treatment.

Temperature had a significant effect on POD activity at the same pressure. With the increase of temperature, POD activity showed an upward trend at 4–40°C, then a downward trend at 60–80°C. Castro et al. (2008) reported similar results, suggesting that thermal blanching treatments caused higher inactivation of POD activity than the pressure treatments. In general, POD is considered to be a thermostable enzyme. However, the heat resistance of POD may be decreased due to the influence of pressure during UHPH treatment.

Similar to the change of PPO activity, the above result could be mainly related to conformational changes of POD during UHPH treatment. With the increase of pressure and temperature, the secondary and tertiary structures of POD had more significant changes, which induced inactivation and activation of POD (Leite Júnior et al., 2021).

## 4 CONCLUSIONS

In summary, UHPH showed the ability to ensure microbiological safety and could maintain the physicochemical properties of composite juice and improve the nutritional properties and suspension stability of the system. Especially, after UHPH treatment at 80°C, the microorganism could not be detected, the total phenols and antioxidant activity were significantly increased, and the enzyme activity was significantly decreased.

Therefore, UHPH may be a better choice for the processing of composite pear juice. However, the activity of endogenous enzymes cannot be completely passivated, so further studies on inactivating enzymes need to be strengthened.

## Supporting information

Appendix S1
