# Supplementary material for: Effect of ultra‐high pressure homogenization on microorganism and quality of composite pear juice
Source: Food Sci Nutr. 2022 Apr 27;10(9):3072–84. doi: 10.1002/fsn3.2906 (PMC9469897; doi:10.1002/fsn3.2906)
Supplement: Supplementary file 1 — Appendix S1 [file FSN3-10-3072-s001.docx]

**S****upplementary Material**

**UHPH instrumentation**

As shown in Figure S1, the equipment is pressurized to the treated material through a high-pressure pump to reach a predetermined pressure and then passes through a homogenization valve. The whole homogenization valve adjusts the temperature through a water bath, and the homogenization valve is cooled or heated to the set temperature in advance during the test, therefore, the outlet and inlet temperatures are the predetermined water bath temperatures. Because of the fixed flow rate and valve design, the holding time is unchanged at 1.15 s.


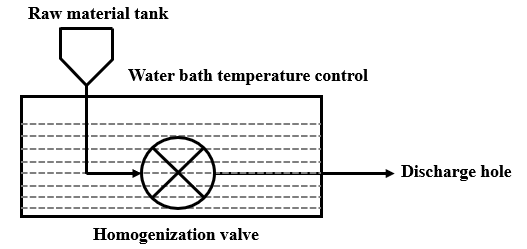


Figure S1 Schematic diagram of the UHPH instrument

Table S1 UHPH instrument parameters

| parameter | average | standard deviation |
| --- | --- | --- |
| the inlet temperature | 4/20/30/40/60/80℃ | 1℃ |
| the outlet temperature | 4/20/30/40/60/80℃ | 1℃ |
| the pressure level | 50/100/150/200 MPa | 5 MPa |
| the holding time | 1.15 s | - |

**Quantification of** **total phenols and antioxidant capacity**










Figure S2 Standard curve of total phenols (a), antioxidant capacity determined by DPPH (b) and FRAP (c)

**Measurement of *D[4,3]* and *D[3,2]***

The statistics of the distribution are calculated from the results using the derived diameters *D[m,n]* – an internationally agreed method of defining the mean and other moments of particle size. It is found that there are V_i_ particles within any size interval i, which has an arithmetic mean diameter of d_i_. When m does not equal n, let any mean diameter *D[m,n]* be represented by:

$$D\left[ m,n \right]=[{\frac{\sum V_{i}d_{i}^{m-3}}{\sum V_{i}d_{i}^{n-3}}]}^{\frac{1}{m-n}}$$
